# Implications and considerations of thermal effects when applying irreversible electroporation tissue ablation therapy

**DOI:** 10.1002/pros.22986

**Published:** 2015-03-23

**Authors:** Rafael V. Davalos, Suyashree Bhonsle, Robert E. Neal

**Affiliations:** ^1^ School of Biomedical Engineering and Sciences Virginia Tech Blacksburg Virginia; ^2^ AngioDynamics Inc Queensbury New York

**Keywords:** IRE, non‐thermal ablation, minimally invasive surgery, focal targeted treatments

## Abstract

Irreversible electroporation (IRE) describes a cellular response to electric field exposure, resulting in the formation of nanoscale defects that can lead to cell death. While this behavior occurs independently of thermally‐induced processes, therapeutic ablation of targeted tissues with IRE uses a series of brief electric pulses, whose parameters result in secondary Joule heating of the tissue. Where contemporary clinical pulse protocols use aggressive energy regimes, additional evidence is supplementing original studies that assert care must be taken in clinical ablation protocols to ensure the cumulative thermal effects do not induce damage that will alter outcomes for therapies using the IRE non‐thermal cell death process for tissue ablation. In this letter, we seek to clarify the nomenclature regarding IRE as a non‐thermal ablation technique, as well as identify existing literature that uses experimental, clinical, and numerical results to discretely address and evaluate the thermal considerations relevant when applying IRE in clinical scenarios, including several approaches for reducing these effects. Existing evidence in the literature describes cell response to electric fields, suggesting cell death from IRE is a unique process, independent from traditional thermal damage. Numerical simulations, as well as preclinical and clinical findings demonstrate the ability to deliver therapeutic IRE ablation without occurrence of morbidity associated with thermal therapies. Clinical IRE therapy generates thermal effects, which may moderate the non‐thermal aspects of IRE ablation. Appropriate protocol development, utilization, and pulse delivery devices may be implemented to restrain these effects and maintain IRE as the vastly predominant tissue death modality, reducing therapy‐mitigating thermal damage. Clinical applications of IRE should consider thermal effects and employ protocols to ensure safe and effective therapy delivery. *Prostate 75:1114–1118, 2015*. © 2015 The Authors. *The Prostate*, published by Wiley Periodicals, Inc.

We read with interest the discussion introduced by your recently published article (van Gemert, Wagstaff, de Bruin, et al. Irreversible Electroporation: Just Another Form of Thermal Therapy? *The Prostate*. 2014). The study used an analytical calculation to approximate cumulative temperature changes encountered during irreversible electroporation (IRE) therapy when delivering pulse protocols commonly employed clinically. The authors conclude that thermal effects are not insignificant, and must be considered when designing effective IRE therapy protocols. We appreciate the authors' comments and their appeal to encourage a forum for discussing the relevance of thermal effects in IRE therapies. These effects cannot be neglected, and have been considerably explored in prior literature. Nonetheless, we agree that continued discussion regarding the relevance and implications of thermal effects is warranted, and would like to take the opportunity to offer clarification and convey relevant findings of previous work. Further, we would like to address some of the claims and statements in their discussion and conclusion, which may inadvertently distort perceptions regarding the utility of IRE therapy, particularly their claim that IRE is not different from Joule heating‐based therapies.

Possible concern regarding the description of IRE as a non‐thermal therapy likely relates to the unresolved need for discretely defining an important clarification. When discussing IRE, there should be an understood distinction between IRE as a biophysical, cellular‐level response to repeated insult from electric field exposure, and IRE as the exploitation of this phenomenon to destroy targeted volumes of tissue therapeutically. When applied appropriately, it is possible to exploit the non‐thermal cell death mechanism to destroy the bulk of tissue affected in IRE therapy without inducing clinically‐relevant thermal damage 1.

## IRE CELL DEATH MECHANISM

At its core, IRE describes cell death that occurs due to irrecoverable defects in the membrane or the chemical imbalances that occur due to the influx and efflux of molecules through transient or stable defects 2, 3. These defects are produced by exposing the cell to an electric field of sufficient magnitude to alter the native transmembrane potential of the cell, lowering the energy thresholds for the formation of hydrophobic and hydrophilic pores 4. The pores have been described by several methods, but were initially identified by monitoring conductivity changes and molecular transport across single cells 5. In practice, they are generated by exposing the cells, both in vitro and in tissue, to a series of brief square wave electric pulses. The pores may resolve following cessation of the pulse, where their transient opening has been used for decades to introduce macromolecules such as chemotherapeutics, 6 and is a routine method for facilitating gene transfection into both prokaryotic and eukaryotic cells 7, 8.

When an electroporation pulse protocol (typically identified by strength of electric field, pulse length, number of pulses, and pulse repetition rate) is of sufficient strength, the cell cannot recover from the insult of the defects and dies. Numerous studies investigate the mechanisms inducing the cell death due to the pulses, using examination of histological morphology and staining characteristics 9, 10, as well as transmission and scanning electron microscopy 11. While there remains discussion regarding the exact pathways in which the cells die, the existing evidence seems to indicate it is a combination of several mechanisms that correspond with energy and field exposure 12, including a rapid‐onset mechanism that gives cells an appearance consistent with cellular necrosis 9, 13, a longer‐term secondary apoptosis 10, 11, 14, and a number of possible tertiary effects when performed in vivo. Such tissue‐level effects may include capillary‐disruption induced edema, temporary vascular occlusion induced hypoxia 15, 16, and immune activation 17, 18. These cell death mechanisms have been recognized in tissue during pulsing protocols where experimental and numerical modeling indicated the influence of the thermal damage process was limited to the immediate region near the electrodes 1, 3, 9, 19. Overall, it is well characterized in the literature, both in vitro and in vivo, that the modality of cell death induced from IRE is discrete and independent from the extent of thermal effects generated.

Because the IRE mechanism induces cell death by affecting the cellular membrane, it is able to kill cells in a targeted region without damaging the collagen and other interstitial tissue constituents, thus preserving the patency of critical structures, which include the major vasculature, neurovascular bundles, ductal systems, or other sensitive tissues such as the urethra. The sparing of these critical structures is a primary distinguishing characteristic for IRE treatments, offering a therapeutic option for targeted tissues that are contraindicated for other local therapies such as surgical resection, thermal ablation, or radiation therapy.

## IRE APPLICATION FOR TISSUE ABLATION

While cell‐scale IRE effects are well described to offer a non‐thermal method for cell death, the application of this modality for therapeutic ablation of targeted tissue volumes can use pulse protocols that will induce some degree and volume of thermal damage within the IRE ablation zone. This is a particular consideration for the high voltage and high pulse number protocols that may be employed when treating large volumes of tissue. Temperature rise results from Joule heating of a conductive medium when exposed to electrical energy, making it an inevitable secondary effect when delivering therapeutic IRE. Thus, it is vital to clarify that the phrasing used to describe non‐thermal IRE therapy does not claim to indicate the complete absence of thermal effects, but rather that their extent and location can be controlled so as to restrict any therapy‐limiting damage, particularly to the vital structures in and around the targeted region, that comprises part of IREs unique focal therapy advantages. When a treatment is described as non‐thermal IRE therapy, it identifies a treatment that exploits the IRE cell death mechanism as the vastly predominant cell death modality for destroying a targeted region of tissue. While some protocols will encounter a limited volume of thermal necrosis, such as that identified in 20, properly applied non‐thermal IRE treatments can maintain sufficiently low temperatures to prevent damage to the vital structures associated with the morbidity and mortality constraints of other focal therapies.

## THERMAL EFFECTS OF IRE THERAPY

Contrary to the previous article's assertions that this has been underexposed in the literature, there is a vast collection of literature that extensively characterizes the extent and implications of thermal effects, both for single pulse protocols, and also for clinically relevant full‐procedure pulse protocols 1, 21, 22, 23, 24, 25, 26. These studies describe the various associated thermal effects possible within the limited regions of greatest temperature change, and offer protocol development guidance 27, 28.

## NUMERICAL EVALUATION OF THERMAL EFFECTS

While numerical simulations offer a low‐risk, cheap, and rapid method for evaluating the thermal implications in IRE therapy, it is important to address their limitations when making claims regarding the distribution and extent of these effects once translated in vivo. The work in the article under discussion provides predicted temperature distributions, similar to existing studies in the literature, but using their own derived equation to determine temperatures reached in tissue. However, their model does not appear to consider several factors that influence these effects when predicting electrical and thermal distributions in living tissue. Such aspects include blood perfusion as a thermal sink, metabolic heat generation, ambient cooling of the electrode, initial temperature of an anaesthetized patient, or temperature behavior of an isolated organ in an open procedure. None of these effects are considered in the authors' published article. Further, the simulation results were not calibrated or referenced to experimental data to evaluate their genuine accuracy. These missing attributes may explain why one of their rapid calculations in the discussion found a predicted temperature rise of 39°C, over twice as high as the 18°C found from the experimental study they reference 20. While this was not their advanced model, it demonstrates the limitations of such simulations. Neglecting in vivo interactions encountered in actual IRE therapy delivery, particularly the time for cooling between pulses in this example, may explain the discrepancy between their calculated results and actual experimental data.

## CLINICAL AND PRECLINICAL EXPERIMENTAL EVALUATION OF THERMAL EFFECTS

Thermal damage to tissue is a time‐dependent process, and is conventionally described by an Arrhenius type equation 29. Contrary to the simulation predictions, experimental, and clinical studies show effective IRE ablation while using actual temperature measurements during delivery to confirm that the ablations occurred without significant thermal effects. This includes a canine sarcoma patient case study 30, and an in vivo canine brain study 31. Temperatures in both cases were monitored immediately adjacent to the electrode, where cumulative thermal effects would be greatest. The brain study confirmed an ablation volume while only showing a maximum temperature rise of 1.15°C. Further, the sarcoma patient showed a temperature rise of only 2.4°C, returning to baseline within 3 min, and resulted in complete remission of the tumor.

In a recent experimental study, a substantial temperature rise was determined for 3‐ and 4‐needle configuration protocols using 70 pulses, each 90 µs long, at a rate of 90 pulses/min 32. The maximum temperatures are found within the core of the ablation 57°C and 79°C for 3‐ and 4‐needle protocols, respectively. Consistent with the rapid decay in thermal effects away from the electrodes, max temperatures of 40 and 42°C were measured 1 cm outside the electrode geometries. Where the study shows ablation extending beyond the pale discoloration regions that suggest thermal damage, it clearly demonstrates the potential superposition of thermal damage within the bulk IRE ablation zone. This recent study indicates the need for careful consideration of thermal effects during IRE ablation therapies, and supports employing pulse delivery protocols and devices to mitigate the extent of thermal effects.

In addition to temperature measurements, extensive in vivo experimental and clinical data outcomes show targeted tissue ablation without morbidity to sensitive structures within ablated regions 32, 33, 34, 35, 36, 37. Thermal therapies risk damage to these structures and cite difficulty in killing cells adjacent to major vasculature due to the thermal sink from blood perfusion. IREs ability to perform effective tissue ablation and attain successful oncologic outcomes in these regions without encountering the complications associated with thermal therapies further asserts the uniqueness of IRE as a distinct ablation modality.

## TECHNIQUES FOR MAINTAINING IRE AS PRIMARY MODALITY FOR TISSUE ABLATION

When large ablation targeted volumes may risk unacceptable levels of thermal effects from IRE therapy, there are several studies in the literature with recommendations on methods to mitigate thermal damage degree and volume for IRE protocols 22, which were not discussed nor incorporated into the authors' simulations. Such approaches include protocol changes using shorter pulse durations, slowing the pulse delivery rate, or delivering small sets of pulses around the pairs of electrodes in a “modulated” delivery approach, which allows more time for conductive tissue cooling between each particular pair of electrodes, with evidence suggesting this may increase ablation zone 38, 39. Additionally, thermal countermeasures have been suggested including the use of actively cooled electrodes, cooling patient baseline temperature, or cooling the region surrounding the target organ with hydrodissection. While the appropriate approach to controlling thermal effects should be considered on a patient‐specific basis, such as more aggressive cooling approaches for more intensive energy delivery or sensitive organs, a combination of these strategies may be ultimately employed to offer IRE without significant thermal damage.

## CONCLUSION

Discussion is valuable and warranted to examine the extent and tolerance of thermal collateral effects that result from IRE therapies. However, this should not be confused with the mechanism of action driving the cell death and outcomes determined from IRE protocols in experimental and clinical models. Effective exploitation of the IRE cell death mechanism for targeted ablation therapies at a tissue‐level can be done with the vast bulk of cell death due to non‐thermal mechanisms. Constraining thermal effects to maintain the unique characteristics of IRE therapy, mainly its ability to spare the critical structures that contraindicate thermal‐based therapies, requires ensuring appropriate treatment protocols are employed. Consideration of appropriately delivered IRE protocols will facilitate bulk tissue treatment with IRE without bulk thermal coagulation, ensuring appreciation of the non‐thermal cell death advantages when IRE is employed in therapeutic tissue ablation.
